# The antimicrobial lysine-peptoid hybrid LP5 inhibits DNA replication and induces the SOS response in *Staphylococcus aureus*

**DOI:** 10.1186/1471-2180-13-192

**Published:** 2013-08-14

**Authors:** Sanne Gottschalk, Dan Ifrah, Sandra Lerche, Caroline T Gottlieb, Marianne T Cohn, Hiroshi Hiasa, Paul R Hansen, Lone Gram, Hanne Ingmer, Line E Thomsen

**Affiliations:** 1 Department of Veterinary Disease Biology, Faculty of Health and Medical Sciences, University of Copenhagen, DK-1870 Frederiksberg, Denmark; 2 Department of Drug Design and Pharmacology, Faculty of Health and Medical Sciences, University of Copenhagen, Copenhagen, DK-2100, Denmark; 3 National Food Institute, Technical University of Denmark, K-2800 Kgs. Lyngby, Denmark; 4 Department of Pharmacology, University of Minnesota Medical School, Minneapolis, MN 55455, USA; 5 Department of Systems Biology, Technical University of Denmark, DK-2800 Kgs. Lyngby, Denmark; 6 Present address: Novo Nordisk, Hagedornsvej, Gentofte, Denmark; 7 Present address: Novo Nordisk Park, Maaloev, Denmark; 8 Present address: Chr. Hansen, Boege Allé, Hoersholm, Denmark; 9 Present address: Novozymes, Krogshoejvej, Bagsvaerd, Denmark

## Abstract

**Background:**

The increase in antibiotic resistant bacteria has led to renewed interest in development of alternative antimicrobial compounds such as antimicrobial peptides (AMPs), either naturally-occurring or synthetically-derived. Knowledge of the mode of action (MOA) of synthetic compounds mimicking the function of AMPs is highly valuable both when developing new types of antimicrobials and when predicting resistance development. Despite many functional studies of AMPs, only a few of the synthetic peptides have been studied in detail.

**Results:**

We investigated the MOA of the lysine-peptoid hybrid, LP5, which previously has been shown to display antimicrobial activity against *Staphylococcus aureus*. At concentrations of LP5 above the minimal inhibitory concentration (MIC), the peptoid caused ATP leakage from bacterial cells. However, at concentrations close to the MIC, LP5 inhibited the growth of *S*. *aureus* without ATP leakage. Instead, LP5 bound DNA and inhibited macromolecular synthesis. The binding to DNA also led to inhibition of DNA gyrase and topoisomerase IV and caused induction of the SOS response.

**Conclusions:**

Our data demonstrate that LP5 may have a dual mode of action against *S*. *aureus*. At MIC concentrations, LP5 binds DNA and inhibits macromolecular synthesis and growth, whereas at concentrations above the MIC, LP5 targets the bacterial membrane leading to disruption of the membrane. These results add new information about the MOA of a new synthetic AMP and aid in the future design of synthetic peptides with increased therapeutic potential.

## Background

Growing concern over the increase in multidrug resistant bacteria has urged the interest for development of new types and classes of antimicrobial compounds. One such class is antimicrobial peptides (AMPs), also known as host defence peptides, that are found in all multicellular organisms and form an important part of the innate immune system [1]. They exhibit antimicrobial activity against a wide range of pathogenic microorganisms, have immune-modulatory effects and enhance the host defence against pathogenic bacteria [2-4]. AMPs are usually small cationic and amphiphatic peptides comprised of less than 40 amino acids with immense diversity in sequence, secondary structure motifs, charge and/or the abundance of certain specific amino acids [5]. Their ability to selectively kill prokaryotic rather than eukaryotic cells, make them promising candidates for drug development. However, one drawback of most natural AMPs as therapeutics is their susceptibility to proteolytic degradation [6]. To overcome this problem an approach known as peptidomimetics has emerged in recent years by which compounds are produced that mimic a peptide structure and/or function but carries a modified backbone and/or non-natural amino acids. The peptide-mimetic compounds have been designed based on essential biophysical characteristics of AMPs: charge, hydrophobicity, and amphiphatic organization [7-9]. Oligomeric N-substituted glycines, also known as peptoids, belong to the simpler AMP-mimetic designs. They are structurally similar to α-amino peptides, but the side chain is shifted to amide nitrogen instead of the α-carbon [10-12]. This feature offers several advantages including protease stability [13], and easy synthesis by the submonomer approach [11].

**Figure 1:**
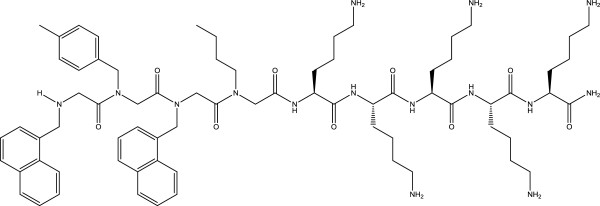
Chemical structure of the lysine-peptoid hybrid LP5.

Previously, a study screening 20 lysine-peptoid hybrids identified a hybrid displaying good antimicrobial activity toward a wide range of clinically relevant bacteria, including *Staphylococcus aureus* (*S*. *aureus*), in addition to low cytotoxicity to mammalian cells [14,15]. The lysine-peptoid hybrid LP5 (lysine-peptoid compound 5) contains the peptoid core [N-(1-naphthalenemethyl)glycyl]-[N-4-methylbenzyl)glycyl]-[N-(1-naphthalenemethyl)glycyl]-N-(butyl)glycin amide and 5 lysines (Figure 1) [14,15]. LP5 is thus potentially interesting as a lead structure in the development of new antimicrobials functioning against pathogens like *S*. *aureus* which are increasingly becoming resistant toward conventional antibiotics [16].

Due to their cationic and amphiphatic nature, it is believed that most AMPs selectively kill bacteria by penetrating the negatively charged cell membrane leading to membrane disintegration. However, during the last two decades it has become apparent that some AMPs may also act by other mechanisms without destruction of the cell membrane, namely, acting on intracellular targets leading to inhibition of enzymatic activities, cell wall synthesis and RNA, DNA and protein synthesis [5,17,18].

The inhibition of RNA, DNA and protein synthesis in bacteria is often the result of AMPs interacting with DNA [19,20]. Additionally, interaction with DNA by the hexapeptide WRWYCR and its D-enantiomers was shown to interfere with DNA repair [21]. DNA repair damage elicits the SOS response that is a conserved pathway essential for DNA repair and restart of stalled or collapsed replication forks, regulated by the repressor LexA and the activator RecA [22,23].

In this study, we set out to investigate the mode of action (MOA) of LP5 using the pathogenic bacterium *S*. *aureus*. Collectively, our data suggest that LP5 has a dual mode of action; at MIC values it is able to bind DNA leading to inhibition of DNA biosynthesis, inhibition of the function of topoisomerase IV (Topo IV) and DNA gyrase and induction of the SOS response through *rec*A. However, when using concentrations above the MIC, LP5 targets the bacterial membrane leading to disruption of the bacterial membrane.

## Results and discussion

### Determination of MIC of LP5 against *S*. *aureus*

Given that the lysine-peptoid LP5 has antimicrobial activity toward a number of bacterial and fungal pathogens, we investigated how LP5 interacts with and affects the pathogenic bacterium *S*. *aureus*. We tested the MIC of LP5 against two *S*. *aureus* strains, 8325–4, a laboratory strain of human origin [24], and the clinically relevant community acquired strain USA300 [25]. MIC was in the range of 16 to 32 μg/ml for both strains.

### Permeabilization of the *S*. *aureus* membrane by LP5 is concentration dependent

**Figure 2:**
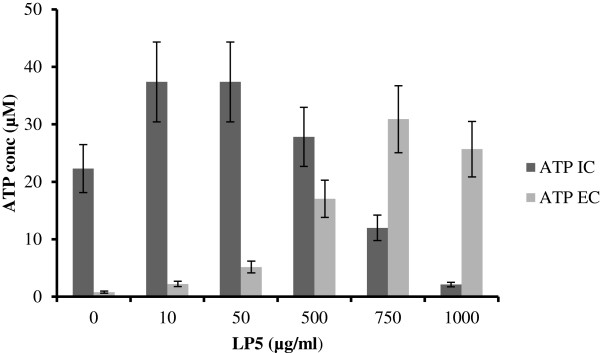
**Measurement of ATP leakage from *S*. *aureus *8325–4 after treatment with LP5.** Measurement of intracellular (IC) and extracellular (EC) ATP after treatment with increasing concentrations of LP5 (0–1000 μg/ml).

Many AMPs interact with the bacterial membrane, leading to pore-formation and subsequently leakage of intracellular components [5]. Therefore, to determine whether LP5 influences *S*. *aureus* membrane structure, we investigated membrane integrity by measuring ATP leakage. We found that increasing concentrations of LP5 added to *S*. *aureus* 8325–4 at time-point 0, lead to a gradual increase in ATP leakage from the cells (Figure 2). The addition of 1000 μg/ml of LP5 most likely resulted in an abrupt destruction of the bacterial membrane, since no intracellular ATP was detectable and an immediate increase in extracellular ATP was detected. However, at low concentrations of LP5 only limited leakage of ATP was observed, showing that the leakage of ATP is concentration dependent. Thus, in this experiment we find that LP5 targets the membrane at high concentrations whereas little effect on the membrane was seen at low concentrations.

**Figure 3:**
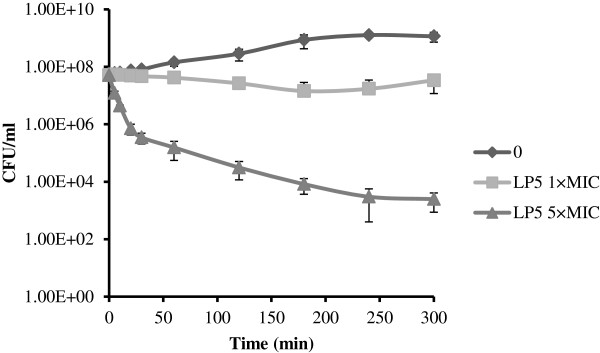
**Kinetics of bacterial killing *in vitro*. ***S*. *aureus* 8325–4 was incubated with LP5 at 0, 1 × MIC or 5 × MIC. CFU, colony-forming units.

These observations agree well with the killing kinetics of LP5 against *S*. *aureus* (Figure 3). Here, we performed dose-dependent time-kill assays at two concentrations representing 1 × MIC and 5 × MIC (Figure 3). LP5 reduced the colony forming unit (CFU) counts by 2 log units during the first 30 min of the experiment at 5 × MIC. Thereafter, the killing rate gradually decreased and after the 5 h time course approached a total reduction of CFU count by 4 log units. At 1 × MIC LP5 did not reduce the CFU within the 5 h of exposure (Figure 3) and the exposed bacteria resumed growth when transferred to media without LP5 (data not shown). Thus, at this concentration LP5 does not to kill *S*. *aureus*, instead it prevents growth, indicating that LP5 does not affect the cell membrane but rather has an intracellular target. This notion is supported by the finding that concentrations several fold above the MIC is needed to see ATP leakage. Some AMPs can cause small membrane lesions, which lead to transient leakage of protons and thereby depletion of intracellular ATP, which would affect synthesis of cellular molecules [26]. However, from our ATP leakage experiment, it is clear that the intracellular level of ATP does not decrease, until high concentrations of LP5 are used and increased ATP leakage is observed (Figure 2).

AMPs have previously been suggested to have multiple targets, including both intracellular targets and the membrane, depending on the concentration of the AMP [18]. Indolicidin and the peptidomimetic oligo-acyl-lysine (OAK) C_12_K-2β_12_ (OAKs: a group of AMPs composed of amino fatty acids) induce membrane damage at magnitudes above their MICs, whereas around their MICs they were both found to have intracellular targets [27-29].

### LP5 inhibits macromolecular synthesis of DNA and binds DNA *in vitro*

**Figure 4:**
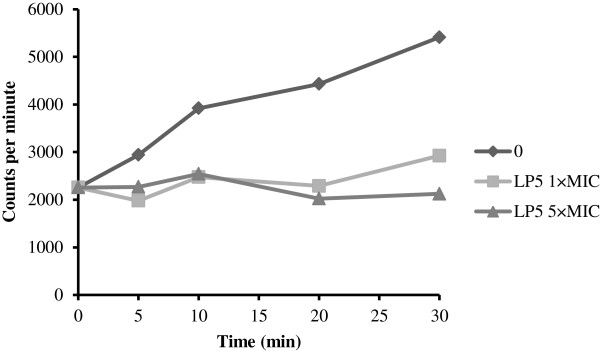
**LP5 inhibit bacterial macromolecular synthesis of DNA.** Effect of LP5 at 1 × MIC and 5 × MIC on DNA synthesis of *S*. *aureus* 8325–4 measured by incorporation of radiolabelled precursors [methyl-^3^H]thymidine. Data are one representative of three independent experiments, which all gave similar results.

AMPs can affect the synthesis of macromolecules [30] and since LP5 is likely to have an intracellular target, we investigated its effect on DNA synthesis. We assessed the ability of *S*. *aureus* to incorporate radiolabeled thymidine into DNA after exposure to concentrations of LP5 at either 1 × MIC or 5 × MIC. The incorporation was monitored over a time period of 30 min and the DNA synthesis was clearly inhibited within the first 5 min after addition of LP5 at both 1 × MIC and 5 × MIC (Figure 4).

**Figure 5:**
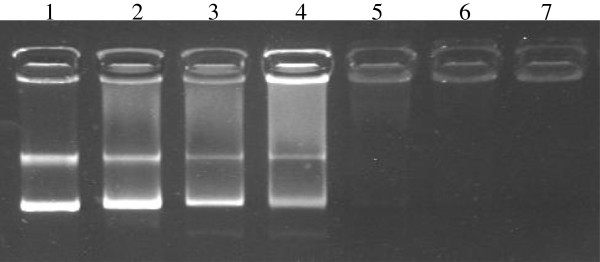
**LP5 binds to DNA.** Gel retardation with *S*. *aureus* DNA. Increasing amounts of LP5 were incubated with 100 ng pRMC2 plasmid DNA and run on an agarose gel. Lane 1: negative control containing binding buffer. Lane 2–7: containing increasing amounts of LP5 (2.5, 5, 10, 20, 40 and 80 μg/ml). The experiment is one representative of four experiments, which all gave similar results.

Previously it has been shown that the inhibition of DNA synthesis by AMPs is associated with their DNA binding [19,20,31]. Therefore, to clarify whether LP5 inhibits DNA synthesis by binding to bacterial DNA, a gel retardation assay was performed. As shown in Figure 5, gel retardation with plasmid DNA demonstrated that in the absence of LP5 pRMC2 migrates as a plasmid. However, upon the addition of increasing concentrations of LP5, the pRMC2 plasmid was no longer able to migrate into the gel. This suggests that LP5 interacts with plasmid DNA and inhibits the migration of plasmid DNA. From the gel retardation assay we observed that at LP5 concentrations well below the MIC value (2.5 μg/ml) LP5 interferes with the migration of plasmid DNA and at 20 μg/ml LP5 the plasmid DNA was altered to such an extent that it no longer entered the gel. DNA binding is not a general property of AMPs, since another peptide, plectasin, did not bind to plasmid DNA in the same experiment (data not shown). The ability of AMPs containing peptoid residues to translocate across lipid bilayers and bind to bacterial DNA has been shown for KLW-L9,13a containing two Nala (Alanine-peptoid) [32]. However, whether this is a general MOA of peptoids still needs to be elucidated. Collectively, these observations strongly support our hypothesis that LP5 exert its MOA intracellularly by binding to DNA and inhibiting DNA synthesis.

### LP5 inhibits DNA gyrase and Topo IV and induces the SOS response through the *rec*A gene

Since LP5 inhibits DNA synthesis and binds DNA we speculated that the DNA replication machinery was affected by LP5. Some of the main players of bacterial DNA replication are the type II topoisomerases, DNA gyrase and Topo IV. DNA gyrase is responsible for the removal of positive supercoils in front of the advancing replication fork, whereas Topo IV decatenates the precatenanes behind the replication fork [33]. To investigate if the activity of these enzymes is influenced by LP5 *in vitro*, supercoiling and decatenation assays were performed using *S*. *aureus* DNA gyrase and Topo IV, respectively. The supercoiling and decatenation activity of *S*. *aureus* DNA gyrase and Topo IV was measured in the presence of various concentrations of LP5 with ciprofloxacin used as a positive control [34].

**Figure 6:**
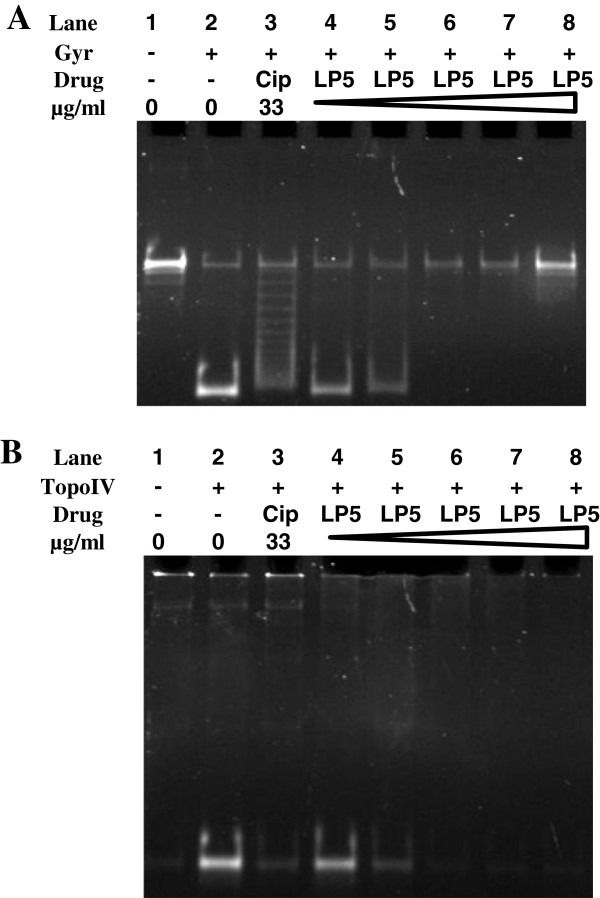
**LP5 affects the supercoiling and decatenation activity of *S*. *aureus *DNA. (A)** The supercoiling reaction mixtures containing relaxed DNA and *S*. *aureus* gyrase (Gyr) (Lane 2–8). Lane 1 served as a negative control containing only relaxed DNA. Lane 3 served as a positive control containing ciprofloxacin (Cip). Lane 4–8 containing increasing concentration of LP5 (66.4 μg/ml to 331.8 μg/ml). **(B)** The decatenation reaction mixtures containing kinetoplast DNA and *S*. *aureus* Topo IV (Lane 2–8). Lane 1 served as a negative control containing only relaxed DNA. Lane 3 served as a positive control containing ciprofloxacin (Cip). Lane 4–8 containing increasing concentration of LP5 (66.4 μg/ml to 331.8 μg/ml).

LP5 was inhibitory on both *S*. *aureus* DNA gyrase and Topo IV in that the enzymes were unable to supercoil or decatenate DNA, respectively (Figure 6). This suggests that LP5 interferes with the activity of both enzymes. However, because we found that LP5 binds to DNA, the observed inhibition of the DNA gyrase and Topo IV is likely due to the inaccessibility of the enzymes to bind to DNA and exert their function possibly leading to stalled replication forks.

**Figure 7:**
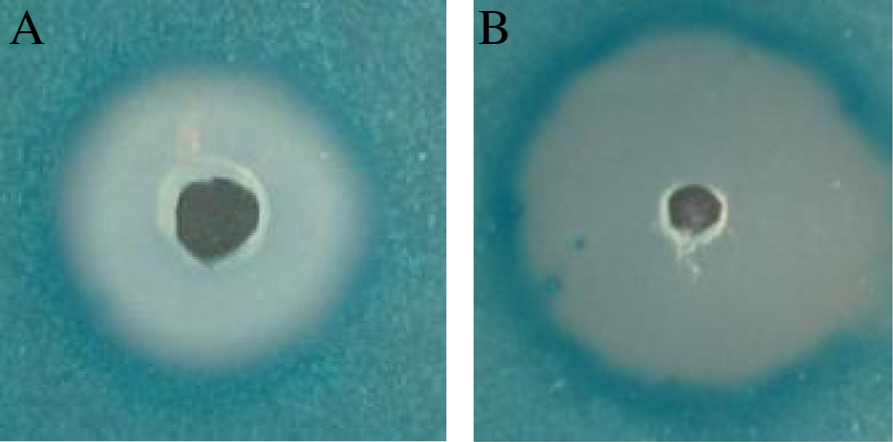
**LP5 induces *rec *A expression in *S*. *aureus*. (A)** LP5 or **(B)** ciprofloxacin (positive control) was added to wells in TSB agar plates containing the *S*. *aureus* 8325–4 derived *lacZ* reporter strain HI2682 (*recA*::*lacZ*). Incubation time was 18 h. Data are one representative of three independent experiments, which all gave similar results.

Stalling of replication forks often lead to induction of the SOS response in bacteria [35]. The ability to induce the SOS response was determined by visualizing the β-galactosidase synthesis from a *recA*-*lacZ* fusion using an agar diffusion assay [36] (Figure 7). The result clearly demonstrated an induction of the expression of *rec*A *in vitro* by LP5 monitored as a blue ring at the point of bacterial growth (Figure 7(A)). Ciprofloxacin was used as a positive control as it is known to induce *rec*A expression in *S*. *aureus* (Figure 7(B)) [37] and H_2_O was used as a negative control (data not shown). The ability to induce the SOS response was shown recently for the hexapeptide WRWYCR that exerts its broad bactericidal activity by inducing the SOS response through stalling of bacterial replications forks [36].

To our knowledge these results show for the first time that a peptoid is able to bind DNA, induce the SOS response and interfere with the functions of DNA gyrase and Topo IV.

## Conclusions

In conclusion, we propose a model in which LP5 exerts a dual MOA. At 1 × MIC the lysine-peptoid hybrid traverses the cytoplasmic membrane of *S*. *aureus* without causing lethal damage and binds the chromosomal DNA, inhibits topo IV and DNA gyrase and thereby the replication machinery by blocking the accessibility to DNA. The inhibitory effect on DNA replication induces the SOS response leading to inhibition of growth. At concentrations of 5 × MIC and above, LP5 also targets the cell membrane leading to leakage of intracellular compounds like ATP, resulting in cell death. These results add new information about the MOA of a new synthetic peptide, and advance our knowledge of these compounds as potential antimicrobial therapeutics.

## Methods

### Peptide synthesis

The synthesis of LP5 was performed using a combination of the sub-monomer approach and Fmoc SPPS, as previously described [38].

### Strains and culture conditions

Three *S*. *aureus* strains were used in this study: Strain 8325–4 [24], FPR3757 USA300 a multidrug resistant community-acquired strain (CA-MRSA) implicated in outbreaks of skin and soft tissue infection [25] and HI2682, which contains a *rec*A-*lacZ* fusion made in this study as described below. The bacteria were grown in Tryptone Soy Broth (TSB, CM0129 Oxoid). When appropriate, antibiotics were added at the following concentrations: 5 and 10 μg/ml tetracycline and 50 μg/ml ciprofloxacin (Sigma).

### Minimum inhibitory concentration determination

The minimum inhibitory concentration (MIC) of LP5 was determined using the modified microtiter broth dilution assay for cationic antimicrobial peptides from Hancock (http://cmdr.ubc.ca/bobh/methods/MODIFIEDMIC.html). Briefly, serial 2- fold dilution of LP5 (at 10 times the required test concentration) was made in 0.2% bovine serum albumin (Sigma, A7906) and 0.01% acetic acid in polypropylene tubes. Overnight cultures of *S*. *aureus* 8325–4 and FPR3757 USA300 were diluted in Mueller Hinton broth (Oxoid, CM0405) to a final concentration of approximately 5 × 10^5^ CFU/ml and 100 μl was added to each well of a 96-well polypropylene microtiter plate. To each well was added 11 μl of the 2-fold serial diluted LP5. The plate was incubated overnight and the MIC was read as the lowest concentration of peptide that inhibited visible growth of *S*. *aureus*. The reported results are from three independent experiments.

### Determination of the effect of LP5 on the bacterial envelope - ATP measurements

Pore formation as caused by peptide addition was determined by measuring ATP leakage from the bacterial cell using a bioluminescence assay as previously described [39]. *S*. *aureus* 8325–4 was grown in TSB at 37°C overnight and then re-inoculated in TSB at 37°C. *S*. *aureus* was harvested (3000 RPM, 10 min) at mid-exponential phase (OD_546_ of 2.5 ± 0.1), washed once in 50 mM potassium phosphate buffer pH 7.0 and once in 50 mM HEPES buffer pH 7.0. The pellet was resuspended in 50 mM HEPES pH 7.0 to a final OD_546_ of 10. Bacteria were stored on ice and used within 5 h. Bacteria were energized in 50 mM HEPES (pH 7.0) with 0.2% (w/v) glucose and treated with various concentrations of LP5 up to a concentration of 1000 μg/ml. ATP measurements were performed at time-point 0. ATP was determined using a bioluminescence kit (Sigma, FLAA-1KT) and a BioOrbit 1253 luminometer. Total ATP content was determined by rapidly permeabilizing 20 μl cell suspension with 80 μl dimethyl sulfoxide. The cell suspension was diluted in 4.9 ml sterile water, and ATP content was determined in 100 μl of the preparation as described by the manufacturer. To determine the extracellular ATP concentration, the 20 μl cell suspension was mixed with 80 μl sterile water and analysed as described above. Intracellular ATP concentrations were calculated by using the intracellular volumes of 0.85 μm^3^. The number of cells in suspension was determined by plate spreading. The reported results are from two independent experiments.

### *In vitro* killing kinetics of *S*. *aureus*

*S*. *aureus* 8325–4 was grown overnight in TSB medium and diluted 1:50 in TSB medium and allowed to grow to OD_600_ of 0.2. LP5 was added to final concentrations equally to one (16 μg/ml) and five times (80 μg/ml) the MIC value, followed by incubation at 37°C while shaking. A control without LP5 was included. At the specified time points aliquots were diluted (serial 10-fold dilutions in saline) and plated on TSB agar. CFU were counted after an overnight incubation at 37°C. The reported results are from three independent experiments.

### Survival of *S*. *aureus* after LP5 exposure

The ability of *S*. *aureus* 8325–4 to resume growth after incubation with 1 × MIC was investigated as follows: bacteria were grown overnight in TSB medium and diluted 1:50 in TSB medium and allowed to grow to OD_600_ of 0.2 LP5 was added to a final concentration 1 × MIC, followed by incubation at 37°C while shaking. A control without LP5 was included. After 3 h cells were harvested and resuspended in fresh TSB medium and 3 × 100 μl cell suspension was added to 100 μl TSB medium in a 96-well microtiter plate. Growth was monitored by measuring the optical density at 600 nm every 20 min for 17 h by the Gen5™ program (BioTek®). The reported results are from two independent experiments.

### Macromolecular synthesis and bacterial killing

Overnight cultures of *S*. *aureus* 8325–4 were diluted 1:50 in TSB and allowed to grow to OD_600_ of 0.2 1 μCi/ml (37MBq) of [methyl-^3^H] thymidine was added to the culture. After 10 min of incubation at 37°C, LP5 was added at 1 × MIC and 5 × MIC. Samples of 500 μl were removed immediately before addition of LP5 (0 min) and at 5, 10, 20 and 30 min after addition of LP5 and added to 2 volume of 99.9% ice cold EtOH and 0.1 volume of 3M sodium acetate (NaAc) pH 5.5 in order to precipitate macromolecules. After overnight precipitation at −20°C samples were collected by centrifugation (12000 rpm, 10 min) and washed twice in 1 ml of ice cold 70% EtOH. Samples were resuspended in 100 μl of milliQ water and added to 4 ml scintillation vials with EcoscintA liquid scintillation cocktail, and counts were obtained in a Beckman scintillation counter for 5 min for each sample using the tritium program. The reported results are from three independent experiments.

### DNA-binding analysis

Gel retardation analysis was performed as previously described [40] by mixing 100 ng of plasmid DNA (pRMC2) [41] isolated from *S*. *aureus* 8325–4 with increasing amounts of LP5 in 20 μl binding buffer (5% w/v glycerol, 10 mM Tris, 1 mM EDTA, 1 mM dithiothreitol, 20 mM KCl and 50 μg/ml bovine serum albumin). Reaction mixtures were incubated 1 h at room temperature and subjected to 1% agarose gel electrophoresis and visualised using ethidium bromide. The reported results are one representative of four independent experiments, showing similar results.

### Construction of *rec*A-*lac*Z fusion

Plasmid pHI1496 carrying a *lac*Z gene was digested with *Sma*I and *Xho*I (New England Biolabs) and ligated into pCL25 (a vector carrying the L54a attachment site for integration into the lipase gene (*geh*) of the *S*. *aureus* chromosome) [42] digested with *Sma*I and *Sal*I (New England Biolabs). The *rec*A promoter was amplified by PCR using the primers RecA-BstBI-F (5′tatttcgaatacggcacctttaccgaaaga3′) and RecA-BamHI-R (5′tatttcgaatacggcacctttaccgaaaga3′) and was cloned into pCR®2.1-TOPO® (Invitrogen)_._ The 663 bp *rec*A sequence was excised from pCR®2.1-TOPO® using *Bst*BI and *Bam*HI (New England Biolabs) and cloned into *Bst*BI/*Bam*HI-cut *lac*-*Z* vector, to give pMTC100 in *E*. *coli* DH5α. The pMTC100 plasmid was electroporated into *S*. *aureus* RN4220 [43] and recombinants selected on 10 μg/ml tetracycline. Since the pMTC100 do not contain a replicon active in *S*. *aureus*, tetracycline resistant clones occur as a result of a recombination event between the plasmid insert and the host. Finally, the *rec*A::*lac*Z fusion from RN4220 was transduced into *S*. *aureus* 8325–4 using the Φ11 phage from *S*. *aureus* 8325 as the carrier [24], and selected on 5 μg/ml tetracycline plates. The expected integrational event was confirmed by colony-PCR using the primers RecA-BamHI-F (5′tatggatcctgacacattaattgagcaagctgt3′) and LexA/lacZ-R (5′cccattcgccattcagg3′). The strain is called HI2682.

### Agar diffusion assay

The assay use a transcriptional reporter strain, HI2682, carrying *lacZ* fused to *recA*. 30 μl of 13.33 mg/ml LP5, 0.05 mg/ml ciprofloxacin or H_2_O was tested in the agar diffusion assay where the expression from the promoter of *rec*A is monitored as previously described [36]. Induction of the *recA* gene was monitored as colour change. The reported results are one representative of three independent experiments, showing similar results.

### Supercoiling and decatenation assays

Supercoiling and decatenation assays were performed as previously described [34] with minor modifications in the reaction mixture content. In the reaction mixtures we used 5 μg/ml tRNA, various concentrations (0; 66.4; 132.7; 199.1; 265.4; 331.8 μg/ml) of LP5 and added either 100 fmol (as a tetramer) of *S*. *aureus* gyrase or 50 fmol of *S*. *aureus* Topo IV. In the control reaction 33 μg/ml ciprofloxacin was used instead of LP5. Additionally, the DNA products were purified with phenol/chloroform to deproteinize the reactions.

## Competing interests

The authors declare that they have no competing interests.

## Authors’ contributions

SG participated in the design of the study, did the experiments and drafted the manuscript, SG and CTG did the ATP leakage analysis. MTC did the HI2682 construction. PRH, SL and DI supplied the Peptoid LP5. SG and HH did the supercoiling and decatenation assays. LET and HI participated in the design of the study and HI, LG and LET helped revise the manuscript. All authors read and approved the final manuscript.
